# A Newsy Letter from San Antonio; the New Medical College, Etc.

**Published:** 1888-05

**Authors:** 


### CORRESPONDENCE.

### A NEWSY LETTER FROM SAN ANTONIO—THE NEW MEDICAL COLLEGE, ETC.

*Editor Daniel's Texas Medical Jouinal:*

I have seen numerous scientific articles in your journal from members of the profession in San Antonio, but do not recollect ever having seen anything in the shape of a "letter" describing what is going on in medical circles in any of our Texas cities.

Now, Mr. Editor, I think an occasional letter from some of the numerous medical gentlemen who write for your most excellent journal, would not only be appropriate, but very interesting. Suppose now when we sit down to write an article for DANIEL'S JOURNAL, instead of writing on "Typhoid Fever," "Pneumonia," "Dysentery," or some of the stereotype diseases of which we are all tired, would it not be a pleasant diversion to describe what is going on in professional circles in our own neighborhoods? I think so, and with your consent propose to take the initiative, if others will follow. I am sure we can make it an interesting feature in the JOURNAL. San Antonio doctors, I know, have been looked upon as a reproach to the medical profession of the State. [News to us.—ED.] Many hard things have been said of us; we have been classed as "fifty cent doctors"; that it was a "stand-off" between us and the mid-wives, in our charges for attending obstetrical cases, etc. While some of this in the past was perhaps true, and there may be a few of the "fifty cent fellows" left, yet of late a very material change has taken place among us, regarding charges.

A few months ago the West Texas Medical Association took the fee bill business in hand, established a code of fees, which was signed by each and every member of the Association; printed copies were furnished for office use, and now I believe the fee bill is adhered to by the great majority of the physicians when the circumstances of the patient will allow his being charged a fair fee.

San Antonio has a population of about 45,000 souls, which is perhaps about equally divided among Americans, Germans and Mexicans. To attend to the medical wants of this population we have about thirty regular physicians, four Homœopaths, and five or six Eclectics and irregulars.

The West Texas Medical Association, perhaps one of the largest local Societies in the State, meets bi-monthly in this city. About sixty members are on its rolls, including those in the city and from adjoining counties. Drs. F. Herff and F. Terrell are respectively President and Secretary.

We have recently welcomed to our professional ranks in San Antonio, Dr. E. Cross, late of Little Rock, Ark. Dr. Cross has for a number of years practiced gynæcology as a specialty. He has formed a co-partnership with Dr. A. Graves, and has established an Infirmary for the treatment of diseases of females, which is meeting with deserved success. Dr. C. is a pleasant gentleman and an accomplished physician, and is quite an accession to our local fraternity.

At the last election held by the Board of Commissioners Dr. P. W. Johns was chosen County Physician. A good selection.

While *en route* to California for the benefit of his wife's health, Dr. Robert Battey, of Rome, Ga., stopped over in San Antonio for a few days to recuperate. Dr. Battey visited our Association and gave a graphic account of Batty's operation and his method of performing it. While in the city Dr. Batty performed, by request, an Oophorectomy, the patient recovering.

The University which is to be established in San Antonio, has become an assured fact, and at the last meeting of the Board of Trustees, a Medical Department was decided upon, and the following gentlemen confirmed as a Faculty :

J. V. Spring, M. D., Dean, Professor Ophthalmology and Otology.

F. Herff, M. D., and Geo. Cupples, M. D., Professors Surgery.

M. K. Taylor, M.D., U. S. A. (Retired), Professor Theory and Practice of Medicine and Hygiene.

T. R. Chew, M. D., Professor Obstetrics.

J. Braunnagel, M. D. Professor Anatomy.

F. Terrell, A. B., M. D., (Harvard,) Professor Physiology.

A. Herff, M. D. Professor Clinical Surgery.

S. T. Lowry, A. M., M. D., Professor Clinical Medicine.

C. E. R. King, M. D., Professor Materia Medica and Therapeutics.

R. Menger, M. D., Professor Pathology and Histology.

E. Cross, M. D., Professor Gynæcology.

P. W. Johns, M. D., Professor Dermatology and Venereal Diseases.

D. Berry, M. D., Demonstor of Anatomy.

E. S. Carothers, M. D., Demonstrator Pathology and Histology.

J. P. Oldham, M. D., Assistant in Gynæcology.

Geo. H. Kalteyer, Professor Chemistry.

Geo. Schmidt, Assistant in Chemistry.

F. Terrell, M. D., Secretary of Faculty.

On account of the length which my letter has already reached, I will refrain from speaking of the relative merits or demerits of San Antonio as a location for a medical school. Suffice it to say, that its Faculty is the strongest that could be gotten together out of any one city in the State, some of its members having a reputation which is not limited by any means to Texas alone.

Its curriculum will be equal to any medical school in the country. Its backers are men of wealth and ability. Clinical advantages will be ample, and with other inducements that the largest city in the State can afford, the Medical Department of the San Antonio University* will be an assured success. More anon.

"MEDICUS."

[*Established under the auspices of the M. E. Church, North. —ED.]

---

MONSTROSITY.—Dr. A. G. Castleman, of Chattanooga, Tenn., reports (*Southern Practitioner*), the birth of a monstrosity to Mr. and Mrs. T., near that city.

The child's bowels, liver, spleen and stomach were developed on the external surface of the abdominal wall and lay to the left of the linea alba, enveloped by a membrane which resembled a mucous membrane, and was continuous with the integument of the abdomen ; the viscera were firmly attached to the external surface of the abdomen, and to the border of the membrane which enclosed them.

The lower limbs were partially deformed. It lived ten minutes. The mother made a good recovery.

